# Integrating age, BMI, and serum N-glycans detected by MALDI mass spectrometry to classify suspicious mammogram findings as benign lesions or breast cancer

**DOI:** 10.1038/s41598-022-25401-0

**Published:** 2022-12-02

**Authors:** Calvin R. K. Blaschke, Elizabeth G. Hill, Anand S. Mehta, Peggi M. Angel, Christine Laronga, Richard R. Drake

**Affiliations:** 1 Department of Cell & Molecular Pharmacology & Experimental Therapeutics, Medical University of South Carolina, Charleston, 29425 USA; 2 Hollings Cancer Center, Medical University of South Carolina, Charleston, 29425 USA; 3 Department of Breast Oncology, H. Lee Moffitt Cancer Center & Research Institute, Tampa, 33612 USA

**Keywords:** Glycobiology, Breast cancer, Diagnostic markers

## Abstract

While mammograms are the standard tool for breast cancer screening, there remains challenges for mammography to effectively distinguish benign lesions from breast cancers, leading to many unnecessary biopsy procedures. A blood-based biomarker could provide a minimally invasive supplemental assay to increase the specificity of breast cancer screening. Serum N-glycosylation alterations have associations with many cancers and several of the clinical characteristics of breast cancer. The current study utilized a high-throughput mass spectrometry workflow to identify serum N-glycans with differences in intensities between patients that had a benign lesion from patients with breast cancer. The overall N-glycan profiles of the two patient groups had no differences, but there were several individual N-glycans with significant differences in intensities between patients with benign lesions and ductal carcinoma in situ (DCIS). Many N-glycans had strong associations with age and/or body mass index, but there were several of these associations that differed between the patients with benign lesions and breast cancer. Accordingly, the samples were stratified by the patient’s age and body mass index, and N-glycans with significant differences between these subsets were identified. For women aged 50–74 with a body mass index of 18.5–24.9, a model including the intensities of two N-glycans, 1850.666 m/z and 2163.743 m/z, age, and BMI were able to clearly distinguish the breast cancer patients from the patients with benign lesions with an AUROC of 0.899 and an optimal cutoff with 82% sensitivity and 84% specificity. This study indicates that serum N-glycan profiling is a promising approach for providing clarity for breast cancer screening, especially within the subset of healthy weight women in the age group recommended for mammograms.

## Introduction

Breast cancer has the highest incidence rate of all cancers, and is the second leading cause of cancer deaths in US women^1^. The stage of the breast cancer at diagnosis has a large impact on the prognosis of the patient, highlighting the need for early detection strategies^1^. Accordingly, the implementation of mammography as a screening tool has decreased breast cancer mortality^2^. While mammograms have a high cancer detection rate, issues of overdiagnosis and false-positives continue to affect their clinical utility^3^. Due to the similar presentations of benign lesions and breast cancer in a mammogram, abnormal interpretations are followed up with additional imaging or a biopsy. A study of over 1.6 million mammograms found that 1.2% of mammograms were classified as suspicious or highly suggestive of malignancy, but after the recommended biopsy, only 71.6% of those patients had breast cancer^4^. With over 39 million mammograms performed in the US every year, this results in over 345,000 unnecessary biopsies yearly in the US alone^5^. To improve breast cancer screening, a supplemental assay is needed to aid in the discrimination of benign lesions and breast cancer.

While mammography is an essential tool in breast cancer screening, issues of low specificity frequently result in unnecessary biopsies or follow-up imaging. These false positives have significant financial costs on a national and individual patient level, as well as a psychological cost for the patient^6–9^. A minimally or non-invasive biomarker for breast cancer detection is needed to increase the specificity of breast cancer screening. Alternative imaging modalities have displayed improvements in cancer detection, especially for women with a higher breast density, but suffer from increased radiation exposure, high costs and low availability, or similar issues with false-positives for digital breast tomosynthesis (DBT), magnetic resonance imaging (MRI), and ultrasound (US), respectively^10–16^. Using blood-based biomarkers for screening benefits from cost-effective and minimally invasive collection. The currently used blood-based biomarkers for breast cancer, such as cancer antigen (CA) 15-3, CA 27.29, carcinoembryonic antigen (CEA), and soluble human epidermal growth factor receptor 2 (sHER2), are primarily used for prognosis, staging, and therapy monitoring, but have limited utility in detection^17–21^. These limitations prompt an investigation into other blood-based biomarkers that can detect differences in the systemic response to breast cancer and benign lesions.

Serum is a rich source of potential biomarkers because of the variety of molecules circulating in the body with altered abundance, activation, and/or composition due to the effects of a disease. Serum biomarkers can be readily adapted into clinical assays because non-invasive collection is cost-effective and large cohorts are available for thorough and accurate analysis of diagnostic potential. The composition of serum collected during blood draws reflects a dynamic biofluid comprised of thousands of proteins, lipids, metabolites, and nucleic acids. A large portion of serum consists of glycosylated proteins produced by the liver and immunoglobulins secreted by B-cells^22^. Glycosylation is a highly regulated metabolic process shown to have essential roles in protein folding, molecular trafficking, protein clearance, and many other processes^23–26^. Being closely aligned with function, altered glycoforms of serum proteins can be dynamic indicators of systemic responses to disease and have been interrogated for potential clinical applications for decades^27–29^.

The creation and optimization of high-throughput techniques for the profiling of serum N-glycans, i.e. a class of glycans linked to a protein via an asparagine residue, has enabled the interrogation of large sample cohorts for robust detection of N-glycan alterations associated with diseases and conditions^30,31^. These methods have been essential for identifying N-glycan alterations associated with clinical factors, such as age and BMI. Age has been characterized by an increase in non-galactosylated serum N-glycans, while BMI is associated with an increase in sialylation of biantennary serum N-glycans^32,33^. Recently, our group has adapted matrix-assisted laser desorption/ionization (MALDI) mass spectrometry techniques for analysis of serum, urine, prostatic fluids, cultured cells, and antibody-captured serum proteins^34–39^. Here, we utilized a high-throughput MALDI mass spectrometry technique^34^ to identify significant differences in the serum N-glycan profiles of 199 women with benign lesions from 99 women with breast cancer. The relative intensity of individual N-glycans and N-glycan classes were compared and evaluated for the ability to discriminate between benign and cancerous conditions, with and without stratification by age and BMI.

## Methods

### Serum samples

Donors were women who reported to the breast surgical clinic at the Moffitt Cancer Center & Research Institute for a breast biopsy after a Breast Imaging Reporting and Data System (BI-RADS) 4 imaging designation. The samples were collected after H. Lee Moffitt Cancer Center & Research Institute institutional review board approval and informed consent from the donors. All experiments were performed in accordance with this protocol following the approved, relevant guidelines and regulations. Serum samples were collected immediately prior to tissue biopsy and deidentified. Age, body mass index (BMI), ethnicity/race, and pathology results of benign lesions (fibroadenomas, fibrocystic, etc.) and breast cancers (invasive or ductal carcinoma in situ (DCIS)) were linked with each specimen. Patients with both an invasive breast cancer and DCIS were treated as invasive. Patients self-identified their ethnicity, and patients identifying as non-Hispanic were asked to self-identify race. The benign samples (n = 199) and breast cancer samples (n = 99) were stratified by age groups according to the current United States Preventative Services Task Force mammogram recommendations (younger than 40, 40–49, 50–74, older than 74) and the established BMI classifications (less than 18.5,18.5–24.9, 25–29.9, more than 29.9)^40^. The clinical characteristics of the samples analyzed in this study are summarized in Table 1.

**Table 1:** Clinical characteristics of samples by diagnosis.

		Benign (n = 199)	Cancer (n = 99)
Ethnicity/Race	Caucasian	151	85
	Hispanic	38	10
	African American	8	1
	Asian	2	3
BMI	Mean ± SD	28.3 ± 5.9	28.7 ± 6.19
	< 18.5	1	2
	18.5–24.9	68	21
	25–29.9	60	42
	> 30	70	34
Age	Mean ± SD	56.7 ± 11.3	57.3 ± 11.9
	< 40	14	7
	40–49	35	17
	50–74	140	68
	> 74	10	7
Pathology	DCIS	–	30
	Invasive	–	69

BMI, body mass index; SD, standard deviation; DCIS, ductal carcinoma in situ.

### Materials

Trifluoroacetic acid (TFA), sodium bicarbonate, and α-cyano-4-hydroxycinnamic acid (CHCA) were purchased from Sigma-Aldrich (St. Louis, MO). Ethanol was purchased from Decon Labs (King of Prussia, PA). Acetonitrile, phosphate buffered saline, HPLC grade water, citraconic anhydride, xylene, methanol, glacial acetic acid, and chloroform were purchased from Fisher Scientific (Hampton, NH). The amine-reactive slides (Nexterion® Slide H) were purchased from Applied Microarray (Tempe, AZ). The attachable well chambers (ProPlate Multi-Array Slide System, 64-well) were purchased from Grace Bio-Laboratories (Bend, OR). Peptide-N-glycosidase F (PNGase F Prime) was purchased from N-Zyme Scientifics (Doylestown, PA).

### Sample preparation

Serum sample preparation was performed as previously described^34^. Serum samples were diluted in sodium bicarbonate and spotted onto an amine-reactive slide. Each slide had spot of PBS diluted in sodium bicarbonate added as a blank. Additionally, a standard healthy serum sample was added to each slide for normalization across slides. All samples and standards were spotted in technical triplicate. The slide was then placed into a humidity chamber for 1 h to bind the serum proteins to the slide. Well chambers were attached to the slide to isolate each sample, and the samples were washed with Carnoy’s solution and water to remove lipids and salts, respectively. After drying the slides, PNGase F was sprayed across the slide with an automated sprayer (M5 TM-Sprayer, HTX Technologies, Chapel Hill, NC) and incubated in a humidity chamber at 37 °C for 2 h to enzymatically cleave the N-glycans from the captured serum glycoproteins. The HTX M5 sprayer was then used to spray CHCA matrix across the slide.

### Mass spectrometry data collection

The slides were imaged with a timsTOF fleX MALDI-QTOF mass spectrometer (Bruker, Billerica, MA) with a SmartBeam 3D laser operating at 10,000 Hz and 20 µm laser spot size. There were 300 laser shots collected per pixel, and a 150 µm raster was used. A 700–4000 m/z range was scanned in positive ion mode.

### Data processing

The mass spectra were imported into SCiLS Lab software (2022b Pro, Bruker, Billerica, MA). Total ion current was used to normalize the images. N-glycan peaks were selected manually using theoretical mass values. Putative structures were assigned to N-glycan peaks based on previously reported assignments^41–43^. N-glycans are reported as m/z values and using the Oxford nomenclature for the putative structures, where A represents the number of antennae present, F indicates the fucose, B indicates the presence of a bisecting *N*-acetylglucosamine, G represents galactoses, and S denotes sialic acids^44^. Multiply sodiated species of the same N-glycan structure were processed, analyzed, and reported individually. N-glycan compositions and mass error are reported in Supplementary Table S1. Maximum peak values were extracted and used for N-glycan absolute intensities. For each slide, the intensity of the blank sample was subtracted from the N-glycan intensity across the samples. N-glycans with less than 2 arbitrary units (au) of intensity after blank subtraction were converted to 1 au to avoid 0 values for the statistical analysis. If a N-glycan was less than 2 au for more than 20% of samples, it was discarded from further analysis. Next, the standard sample was used to create normalization factors for each N-glycan on each slide, where the intensity of the individual slide’s standard was divided by the average intensity of the standards across all slides. Each slide’s N-glycans were then multiplied by the corresponding normalization factor. The intensities were then converted to relative intensities by dividing individual N-glycan intensities by the summed intensities of all the N-glycans in that sample. N-glycan class intensities were calculated by summing the relative intensities of the N-glycans containing the structural or compositional traits of the N-glycan class.

### Statistics

Linear regression was used to model log-transformed N-glycan relative intensities as a function of disease status, controlling for age, ethnicity/race, and BMI. Logarithmic transformation was used to induce approximate normality and stabilize variance. Differences in log-relative intensities across disease status levels were evaluated using model-based contrasts. A 5% false-discovery rate was used to adjust for test multiplicity^45,46^. A similar modeling approach was used to evaluate the association between N-glycan intensities and age or BMI. Loess smoothing was used for graphical exploration of the functional relationship prior to modeling^47^. The performance of N-glycans to classify disease status was evaluated using the area under the receiver operating characteristic curve (AUROC) of a logistic regression model of the patient pathology as a function of the selected N-glycans, N-glycan classes, and clinical characteristics. AUROCs are reported with 95% confidence intervals. Optimal sensitivity and specificity were defined as the sensitivity and specificity of the threshold at the cutoff point of the curve closest to (0,1). All statistical analyses were performed using R version 4.1.0.

## Results

**Figure 1:**
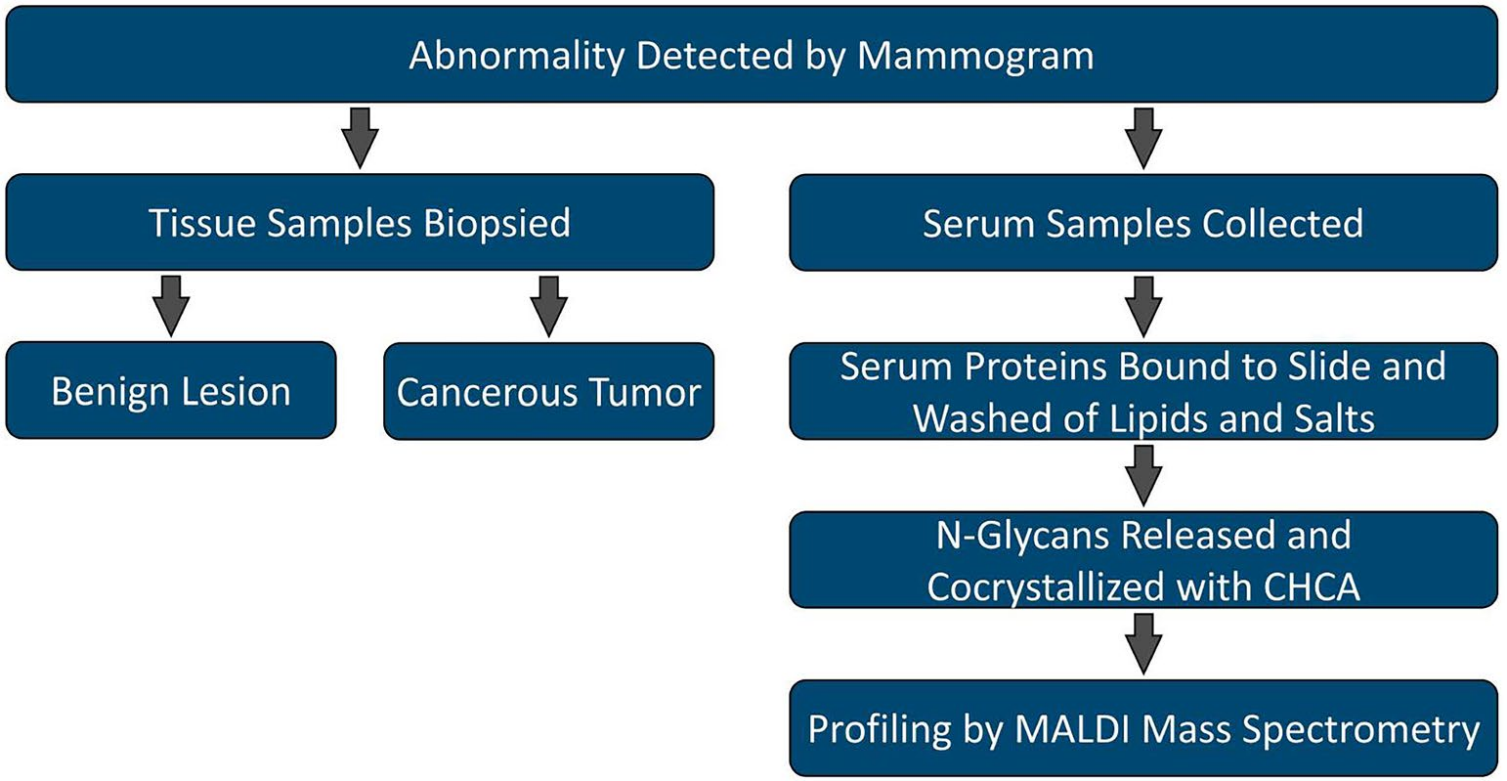
Sample collection, processing, and analysis overview. MALDI, matrix-assisted laser/desporption ionization; CHCA, α-cyano-4-hydroxycinnamic acid.

Serum N-glycan profiles of women with either a benign lesion or breast cancer detected after a mammogram were determined by MALDI mass spectrometry of enzymatically cleaved N-glycans from serum glycoproteins captured and washed on an amine-reactive slide as previously described (Fig. 1)^34^. After data processing and application of data quality criteria, 55 N-glycans, including 10 multiply sodiated N-glycans, were detected across the samples with an average coefficient of variation (CV) of 7.9% across the technical triplicates (Supplementary Table S1). The clinical descriptions of the patients included ethnicity/race, age, and BMI, and had no significant differences between the benign and cancerous samples (Table 1).

**Figure 2:**
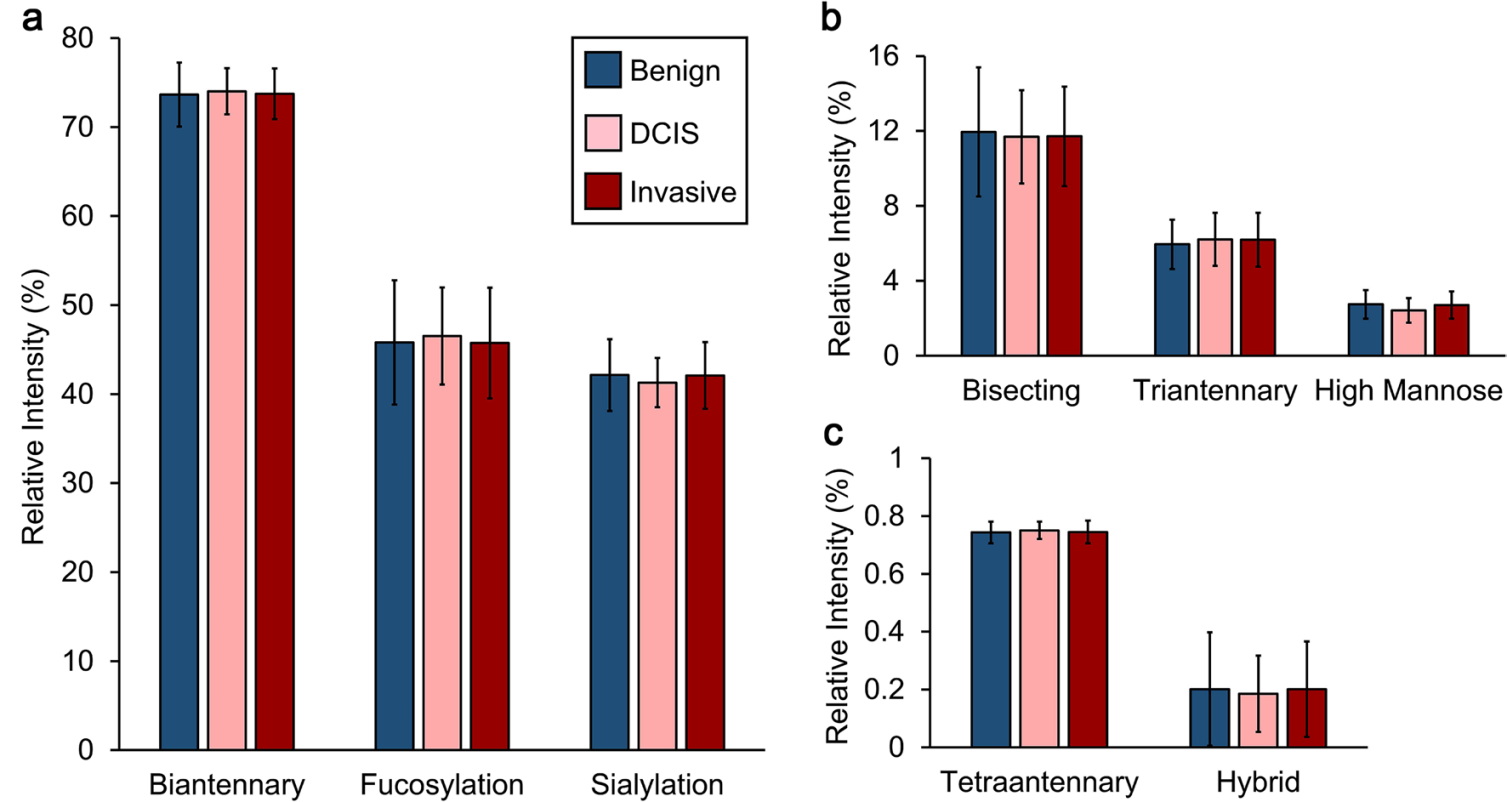
N-glycan class intensities of serum samples with benign lesions, DCIS, and invasive breast cancer. (**a**) High, (**b**) medium, and (**c**) low intensity serum N-glycan classes of benign, DCIS, and invasive samples. Data was analyzed by linear regression modeling of log-transformed relative intensities controlled for age, ethnicity/race, and BMI to identify differences across disease status. Using a 5% false-discovery rate, no significant associations were found.

**Figure 3:**
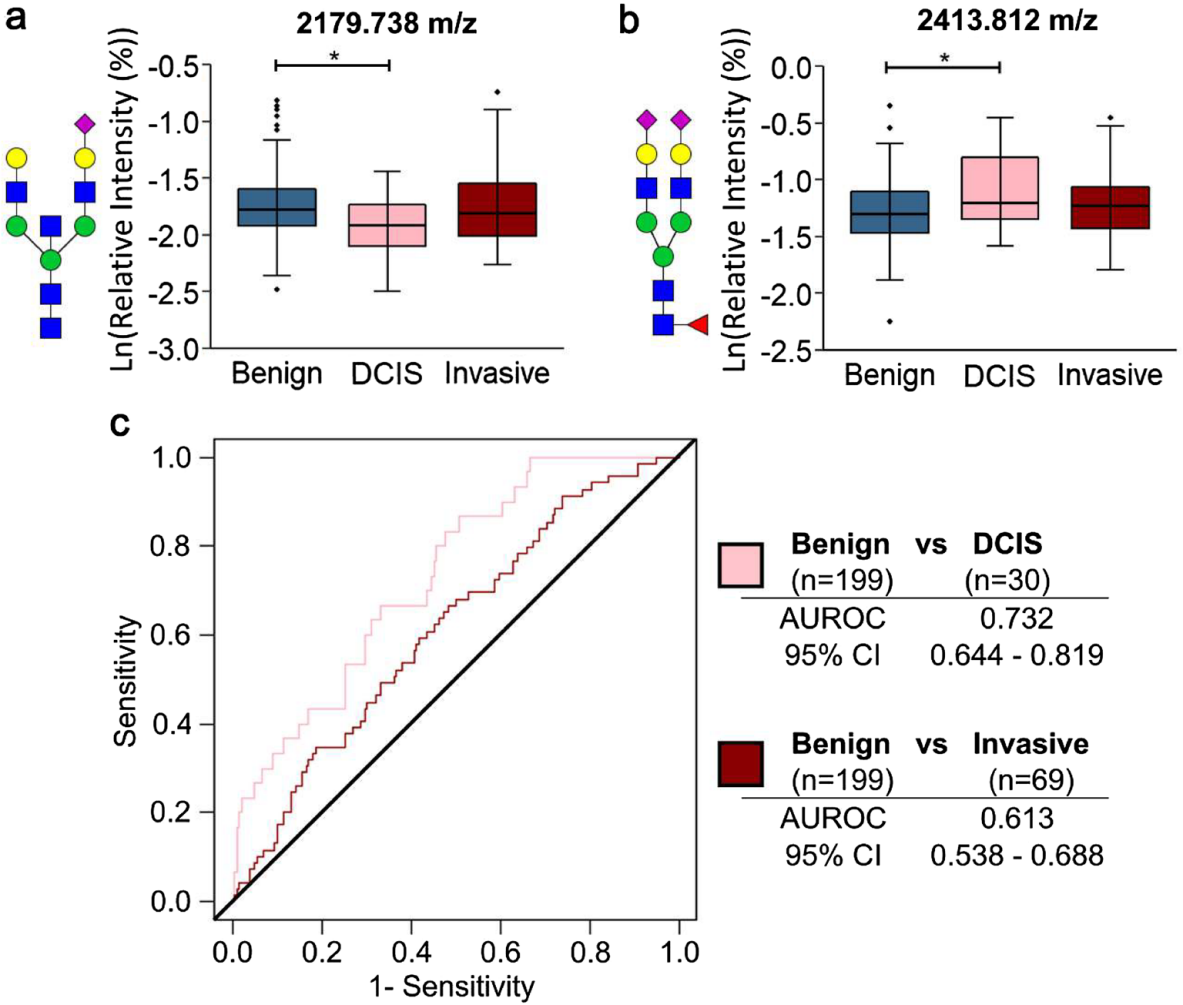
Serum N-glycan differences between benign, DCIS, and invasive samples. Log-transformed relative intensity values of the (**a**) 2179.738 m/z (A2BG2S1) and (**b**) 2413.812 m/z (A2G2F1S2) N-glycans had significant differences in benign and DCIS samples using a 5% false discovery rate. (**c**) Using the log-transformed relative intensities of these N-glycans, age, BMI, and ethnicity/race, benign and DCIS samples could be distinguished with an AUROC of 0.732, and benign and invasive samples could be distinguished with an AUROC of 0.613. N-glycan compositions are represented by blue squares for N-acetylglucosamine, green circles for mannose, yellow circles for galactose, purple diamonds for sialic acid, and red triangles for fucose.

As the clinical characteristics and prognosis of DCIS and invasive breast cancer vary greatly, initial analysis was focused on detecting differences between benign, DCIS, and invasive breast cancer. No differences were detected between the N-glycan classes, with the majority of the N-glycan profile intensity being attributed to biantennary, fucosylated, and/or sialylated N-glycans (Fig. 2). As no differences in the overall N-glycan structural class profiles were detected, individual N-glycan intensities were investigated. There were two N-glycans (2179.738 m/z (A2BG2S1) and 2413.812 m/z (A2G2F1S2)) with significantly different intensities in the benign and DCIS serum samples (Fig. 3, Supplementary Table S2). The 2179.738 m/z N-glycan has a bisected and sialylated structure and had a lower intensity in the DCIS samples. The 2413.812 m/z N-glycan has a fucosylated and multiply sialylated biantennary structure, and had a higher intensity in the DCIS samples (Fig. 3a, b). Based on a logistic model using the intensities of these two N-glycans, age, ethnicity/race, and BMI, an AUROC of 0.732 (0.644–0.819) was found for the discrimination of benign and DCIS samples (Fig. 3c). Using the same variables, an AUROC of 0.613 (0.538–0.688) was found for the discrimination of benign and invasive samples (Fig. 3c). For the evaluation of the entire set based on clinical designation, there were no N-glycan classes or individual N-glycans with differences between the benign and invasive or benign and all cancerous samples.

**Figure 4:**
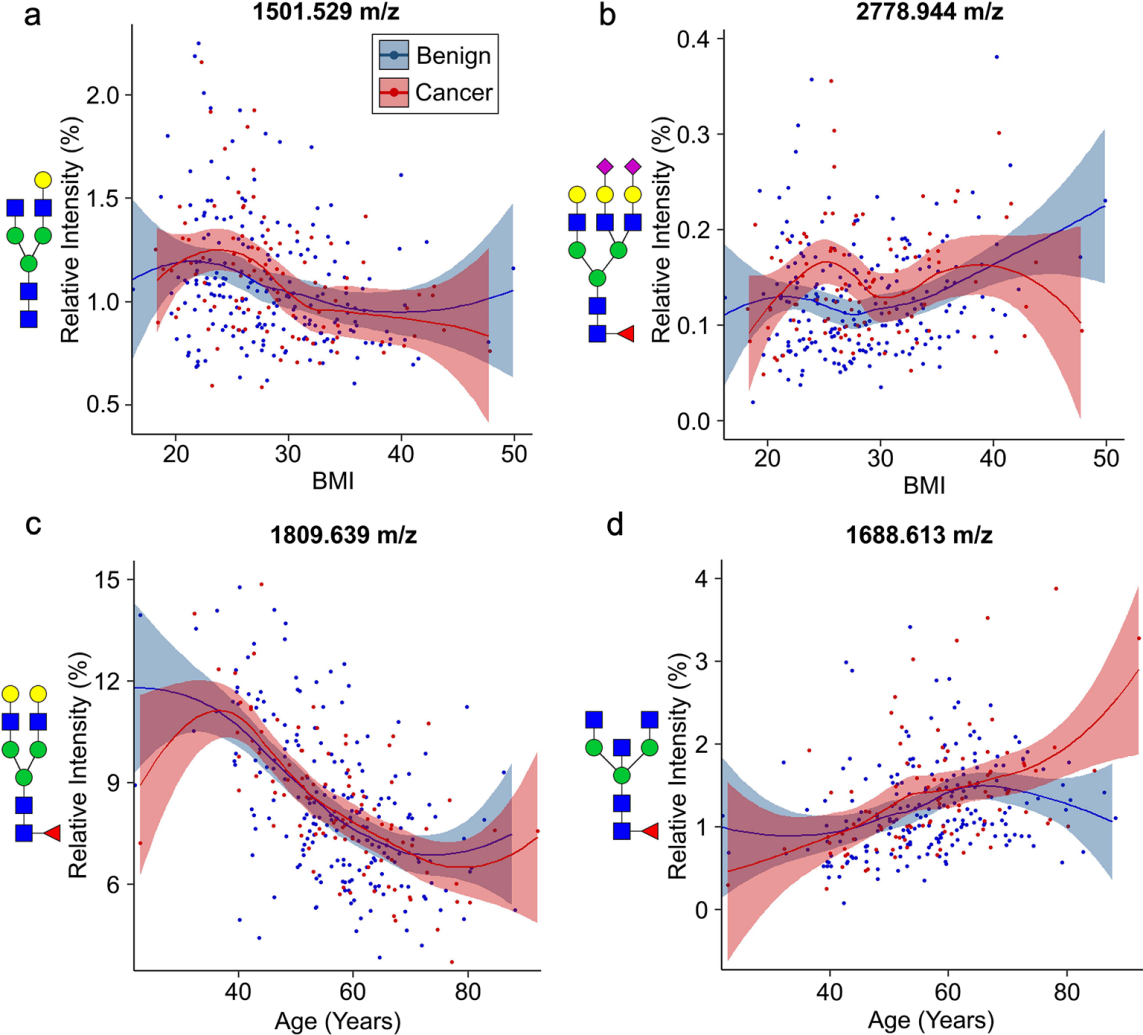
Correlation of serum N-glycan intensities with the patient age and BMI. The patient BMI and the intensities of both (**a**) 1501.529 m/z (A2G1) and (**b**) 2778.944 m/z (A3G3F1S2) N-glycans, but the 2778.944 m/z N-glycan and BMI association diverged for benign and cancer samples in the patients with a BMI from 20 to 30. The patient age and the intensities of both (**c**) 1809.639 m/z (A2G2F1) and (**d**) 1688.613 m/z (A2BF1) N-glycans were significantly associated, but the 1688.613 m/z N-glycan and age association diverged for benign and cancer samples in the older patients. N-glycan compositions are represented by blue squares for N-acetylglucosamine, green circles for mannose, yellow circles for galactose, purple diamonds for sialic acid, and red triangles for fucose.

Additional analysis identified significant relationships between N-glycan intensities and patient age or BMI. There were 24 N-glycans associated with age, as well as the triantennary, tetraantennary, and fucosylation N-glycan classes (Supplementary Table S3). Additionally, there were 12 N-glycans associated with BMI, as well as the biantennary, triantennary, and tetraantennary N-glycan classes (Supplementary Table S4). While some of these associations were present in both benign and cancerous samples (Fig. 4a, c), others were only present in benign samples or displayed differences between the samples at certain ranges (Fig. 4b, d). These findings prompted an in-depth analysis of the samples stratified by age and BMI.

**Figure 5:**
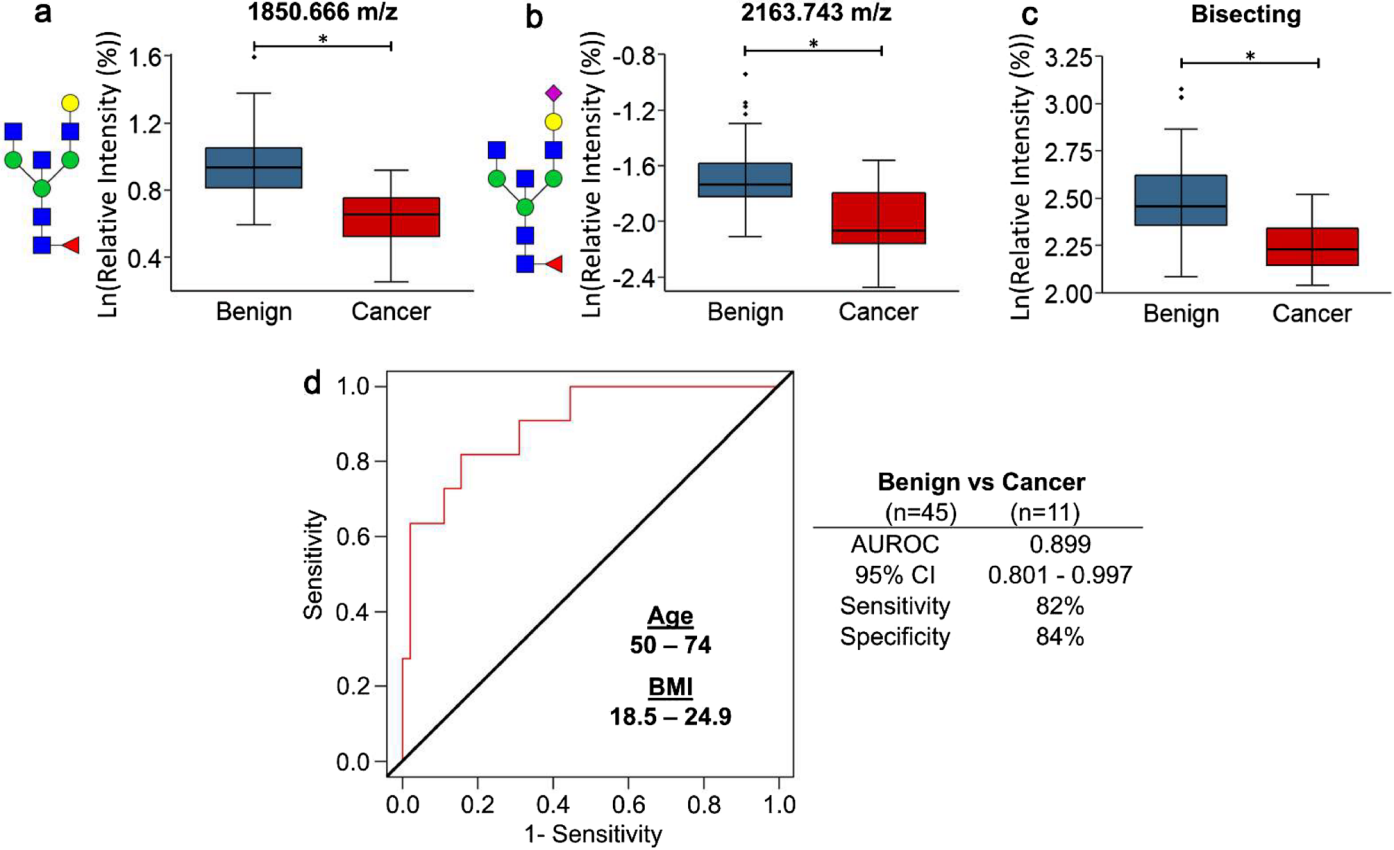
Serum N-glycan differences between benign (n = 45) and cancer (n = 11) samples from patients aged 50–74 and with a BMI of 18.5–24.9. Log-transformed relative intensity values of the (**a**) 1850.666 m/z (A2BG1F1) and (**b**) 2163.743 m/z (A2BG1F1S1) N-glycans and (**c**) bisecting N-glycan class had significant differences in benign and cancer samples using a 5% false discovery rate. (**d**) Using the log-transformed relative intensities of the two individual N-glycans, age, and BMI, benign and cancer samples could be distinguished with an AUROC of 0.899, sensitivity of 82%, and specificity of 84%. N-glycan compositions are represented by blue squares for *N*-acetylglucosamine, green circles for mannose, yellow circles for galactose, purple diamonds for sialic acid, and red triangles for fucose.

The samples were separated by age (less than 40, 40–49, 50–74, and older than 74) and/or by BMI (less than 18.5, 18.5–24.9, 25–30, and more than 30). There were no significant findings between the benign and cancerous samples in any individual or combination of these groups, except for the patients that were 50–74 years old and within 18.5–24.9 BMI. These samples had significant differences in intensity between benign (n = 45) and cancerous samples (n = 11) for two N-glycans with, 1850.666 m/z (A2BG1F1) and 2163.743 m/z (A2BG1F1S1), and the bisecting N-glycan class (Fig. 5a–c, Supplementary Table S5). Based on a logistic model using the intensities of 1850.666 m/z and 2163.743 m/z, age, and BMI an AUROC of 0.899 (0.801–0.997) found for the discrimination of benign and cancerous samples with an 82% sensitivity and 84% specificity (Fig. 5d).

## Discussion

In this study, the serum N-glycan profiles of patients with an abnormality detected by a mammogram were compared to evaluate their ability to distinguish benign lesions from breast cancer. The samples were processed using a recently developed high-throughput MALDI mass spectrometry workflow^34^. When analyzing all the samples, two N-glycans had significant differences in the benign and DCIS samples. Incorporating the intensities of these N-glycans with age, ethnicity/race, and BMI had moderate diagnostic potential for samples from patients with DCIS but not from patients with invasive breast cancer. There were many N-glycan intensities that had altered associations with age or BMI, depending on the diagnosis of the patient. Stratifying the patients by age and BMI identified two N-glycans and two N-glycan classes with significant intensity differences in the samples from patients with benign lesions and breast cancer aged 50–74 with a BMI of 18.5–24.9, and when combined with age and BMI had high diagnostic ability for this patient subset.

Inflammatory stimuli due to the presence of a disease can cause an alteration in the glycosylation of plasma cells and hepatocytes, which produce the bulk of serum proteins^48,49^. Altered serum N-glycan profiles have been associated with a range of diseases and conditions, including Crohn’s disease, gastric cancer, ovarian cancer, Alzheimer’s disease, and hepatocellular carcinoma^50–54^. Serum analysis of breast cancer patients have identified N-glycan changes associated with circulating tumor cell counts, breast cancer subtypes, prognosis, breast cancer progression, and lymph node metastasis^55–60^. While several studies have investigated breast cancer detection by serum N-glycan analysis, they have either had healthy women donors as controls, used methods unable to identify individual N-glycans, or only analyzed certain types of N-glycans^61–63^. To properly address the issue of false-positives in mammograms, the samples from patients with breast cancer need to be compared to samples from patients with a lesion detected by a mammogram that requires a biopsy, because healthy women with no abnormalities detected by mammogram do not suffer from the issues of false-positives. This cohort can appropriately investigate the potential of serum N-glycan analysis for breast cancer detection because of the clinical relevance of the samples collected from women with benign lesions, the controlled surgical collection site, and the availability and incorporation of age, ethnicity/race, and BMI into the analysis.

Age and BMI have strong associations with breast cancer risk, as well as specific alterations in the serum N-glycan profile^29,64–66^. In this study, there were 24 N-glycans with significant associations with age, and 12 N-glycans with significant associations with BMI (Supplementary Tables S3 and S4). Age and BMI were controlled for in the initial analysis to account for these associations and isolate N-glycan intensity differences that were due to the presence of breast cancer. Further investigation found that only samples within certain ranges of age and BMI had disease-associated serum N-glycan changes (Fig. 4b–d). To identify any disease-associated serum N-glycan intensities changes only present in a subset of the cohort, the samples were stratified according to age and BMI. To remain clinically relevant, the samples were grouped based on the USPSTF mammogram recommendation for age and the BMI classifications for underweight, healthy weight, overweight, and obese^40^.

The subset of samples from patients with a BMI of 18.5–24.9 and aged 50–74 could be readily distinguished as having a benign lesion or breast cancer using the intensities of two N-glycans, age, and BMI (Fig. 5d). While this is a subset of the population, it is makes up a significant portion of the U.S. women that will have mammogram screening for breast cancer. For women with an average-risk of breast cancer, the USPSTF recommends only women aged 50–74 to have biennial mammography screening and make up the bulk of women that will have a mammogram^40^. Results from the 2017–2018 National Health and Nutrition Examination Survey (NHANES) indicated that approximately one third of U.S. adult women are within the 18.5–24.9 BMI range^67^. The healthy weight women may have had the best N-glycan biomarker performance due to the lack of systemic inflammation that is present in overweight and obese women^68^. The plasma cells and hepatocytes, from which the majority of serum glycoproteins originate, undergo significant cellular changes in presence of inflammatory stimuli. The serum N-glycan changes found in cancer patients may be masked, altered, or negated by the N-glycan changes due to the obesity-induced inflammatory stimuli.

There were several limitations to this study. Breast cancer is an incredibly heterogeneous disease, that includes multiple molecular and genomic subtypes with distinct clinical behavior and biological characteristics: luminal-A, luminal-B, HER2-positive, and basal-like. The risk factors for breast cancer, including age, body mass index (BMI), onset of menopause, parity, family-history, and personal history of benign breast disease differ in effect for each molecular subtype^69^. As subtype information was not available for this cohort, there may be N-glycan alterations only in certain subtypes that are not being detected. Additionally, there were fewer samples from Asian or African American patients. This makes it more difficult to account for variances due to ethnicity/race, especially after stratifying the cohort into smaller subsets. In the 11 breast cancer patients who were aged 50–74 and within the 18.5–24.9 BMI range, 4 had DCIS and 7 had invasive breast cancer. While both breast cancer groups are represented in these samples, future studies with more patients to represent this subset of women can solidify these findings.

Additional studies identifying the protein(s) that the distinctive N-glycans originated from could increase the performance of the N-glycans as breast cancer detection biomarkers. Recent glycoproteomic approaches have made high-throughput analysis of complex biological fluids quicker and more sensitive^70^. Glycopeptide analysis of this cohort could identify the proteins that contain the cancer-associated N-glycans identified in this study. Alternatively, a technique using MALDI mass spectrometry to N-glycan profile antibody-captured serum proteins could be used to identify breast cancer-associated N-glycan alterations across an array of targets^39,71^. Isolating low-abundant proteins shed from breast cancer cells or released from dying cancer cells could identify N-glycan changes with a higher specificity across all age and BMI ranges. To further investigate the fucosylated bisecting N-glycans that had a significant association with breast cancer, the location of the fucose could be determined by applying Endoglycosidase F3 to the samples instead of PNGase F to detect only core fucosylated N-glycans^34^.

In summary, a MALDI mass spectrometry approach was utilized to detect serum N-glycans and N-glycan classes that could distinguish patients with an abnormal mammogram result as having a benign lesion or breast cancer. For a significant portion of the women screened by mammogram, fucosylated bisecting N-glycans could readily distinguish patient groups.

## Supplementary Information

Supplementary Tables.

## Data Availability

The datasets generated during and/or analyzed during the current study are available from the corresponding author on reasonable request.
