# Supplementary material for: Integrating age, BMI, and serum N-glycans detected by MALDI mass spectrometry to classify suspicious mammogram findings as benign lesions or breast cancer
Source: Sci Rep. 2022 Dec 2;12:20801. doi: 10.1038/s41598-022-25401-0 (PMC9718781; doi:10.1038/s41598-022-25401-0)
Supplement: Supplementary file 1 — Supplementary Tables. [file 41598_2022_25401_MOESM1_ESM.pdf]

Supplementary Table 1. Peak list of N-glycans detected in serum samples. N-glycan compositions are represented by blue squares for N-acetylglucosamine, green circles for mannose, yellow circles for galactose, purple diamonds for sialic acid, and red triangles for fucose.

Theoretical m/z	Observed m/z	Error (ppm)	Composition	Putative Structure	Oxford Nomenclature	Glycosylation Traits	Coefficient of Variation (%)
1136.396	1136.399	2.64	Hex3HexNAc3 + Na1	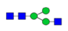	A1		5.69
1257.423	1257.424	0.72	Hex5HexNAc2 + Na1	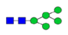	M5	High Mannose	7.76
1282.454	1282.453	0.86	Hex3dHex1HexNAc3 + Na1	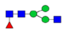	A1F1	Fucosylated	9.37
1298.449	1298.455	4.16	Hex4HexNAc3 + Na1	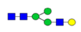	A1G1		6.28
1339.476	1339.478	1.49	Hex3HexNAc4 + Na1	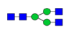	A2	Biantennary	8.09
1419.476	1419.479	2.32	Hex6HexNAc2 + Na1	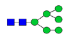	M6	High Mannose	9.98
1444.507	1444.509	1.59	Hex4dHex1HexNAc3 + Na1	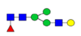	A1G1F1	Fucosylated	5.97
1485.534	1485.535	0.74	Hex3dHex1HexNAc4 + Na1	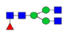	A2F1	Fucosylated, Biantennary	7.79
1501.529	1501.530	1.07	Hex4HexNAc4 + Na1	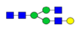	A2G1	Biantennary	6.45
1611.527	1611.523	2.23	Hex4HexNAc3NeuAc1 + Na2	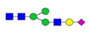	A1G1S1	Sialylated	5.98
1622.555	1622.551	2.22	Hex6HexNAc3 + Na1	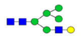	M5A1G1	Hybrid	7.03
1647.587	1647.592	3.22	Hex4dHex1HexNAc4 + Na1	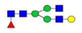	A2G1F1	Fucosylated, Biantennary	7.41
1663.581	1663.585	1.98	Hex5HexNAc4 + Na1	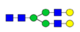	A2G2	Biantennary	5.22
1688.613	1688.617	2.25	Hex3dHex1HexNAc5 + Na1	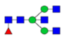	A2BF1	Fucosylated, Bisected	8.67
1757.585	1757.583	0.74	Hex4dHex1HexNAc3NeuAc1 + Na2	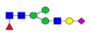	A1F1G1S1	Fucosylated, Sialylated	7.10
1809.639	1809.643	2.16	Hex5dHex1HexNAc4 + Na1	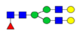	A2F1G2	Fucosylated, Biantennary	4.99
1850.666	1850.674	4.16	Hex4dHex1HexNAc5 + Na1	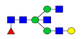	A2BF1G1	Fucosylated, Bisected	6.42
1866.661	1866.667	3.48	Hex5HexNAc5 + Na1	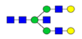	A2BG2	Bisected	6.58
1935.632	1935.634	0.67	Hex6HexNAc3NeuAc1 + Na2	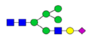	M5A1G1S1	Hybrid, Sialylated	7.69
1938.682	1938.685	1.34	Hex4dHex1HexNAc4NeuAc1 + Na1	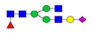	A2F1G1S1	Fucosylated, Sialylated, Biantennary	8.92
1954.677	1954.676	0.20	Hex5HexNAc4NeuAc1 + Na1	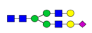	A2G2S1	Sialylated, Biantennary	9.06
1955.697	1955.683	7.11	Hex5dHex2HexNAc4 + Na1	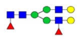	A2F2G2	Fucosylated, Biantennary	8.03
1960.664	1960.669	2.65	Hex4dHex1HexNAc4NeuAc1 + Na2	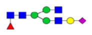	A2F1G1S1	Fucosylated, Sialylated, Biantennary	6.34
1976.659	1976.662	1.72	Hex5HexNAc4NeuAc1 + Na2	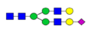	A2G2S1	Sialylated, Biantennary	4.78

2012.719	2012.725	3.23	Hex5dHex1HexNAc5 + Na1	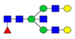	A2BF1G2	Fucosylated, Bisected	6.02
2028.714	2028.719	2.71	Hex6HexNAc5 + Na1	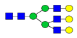	A3G3	Triantennary	9.04
2100.735	2100.740	2.48	Hex5dHex1HexNAc4NeuAc1 + Na1	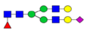	A2F1G2S1	Fucosylated, Sialylated, Biantennary	8.69
2122.717	2122.723	2.87	Hex5dHex1HexNAc4NeuAc1 + Na2	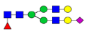	A2F1G2S1	Fucosylated, Sialylated, Biantennary	5.57
2157.756	2157.790	15.71	Hex5HexNAc5NeuAc1 + Na1	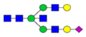	A2BG2S1	Sialylated, Bisected	6.24
2163.743	2163.743	0.09	Hex4dHex1HexNAc5NeuAc1 + Na2	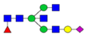	A2BF1G1S1	Fucosylated, Sialylated, Bisected	7.09
2174.772	2174.774	1.01	Hex6dHex1HexNAc5 + Na1	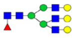	A3F1G3	Fucosylated, Triantennary	6.75
2179.738	2179.738	0.18	Hex5HexNAc5NeuAc1 + Na2	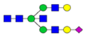	A2BG2S1	Sialylated, Bisected	6.96
2267.754	2267.753	0.38	Hex5HexNAc4NeuAc2 + Na2	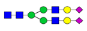	A2G2S2	Sialylated, Biantennary	7.34
2287.819	2287.770	21.72	Hex4dHex2HexNAc5NeuAc1 + Na1	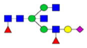	A2BF2G1S1	Fucosylated, Sialylated, Bisected	8.09
2289.736	2289.743	3.01	Hex5HexNAc4NeuAc2 + Na3	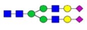	A2G2S2	Sialylated, Biantennary	7.24
2303.814	2303.822	3.52	Hex5dHex1HexNAc5NeuAc1 + Na1	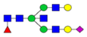	A2BF1G2S1	Fucosylated, Sialylated, Bisected	9.76
2319.809	2319.808	0.26	Hex6HexNAc5NeuAc1 + Na1	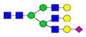	A3G3S1	Sialylated, Triantennary	10.75
2325.796	2325.801	2.19	Hex5dHex1HexNAc5NeuAc1 + Na2	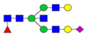	A2BF1G2S1	Fucosylated, Sialylated, Bisected	6.45
2341.791	2341.799	3.54	Hex6HexNAc5NeuAc1 + Na2	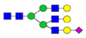	A3G3S1	Sialylated, Triantennary	8.75
2377.851	2377.857	2.48	Hex6dHex1HexNAc6 + Na1	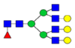	A4F1G3	Fucosylated, Tetraantennary	9.21
2393.846	2393.854	3.38	Hex7HexNAc6 + Na1	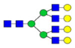	A4G4	Tetraantennary	10.61
2413.812	2413.818	2.28	Hex5dHex1HexNAc4NeuAc2 + Na2	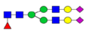	A2F1G2S2	Fucosylated, Sialylated, Biantennary	8.50
2435.794	2435.798	1.77	Hex5dHex1HexNAc4NeuAc2 + Na3	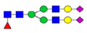	A2F1G2S2	Fucosylated, Sialylated, Biantennary	6.85
2471.854	2471.857	1.09	Hex5dHex2HexNAc5NeuAc1 + Na2	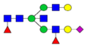	A2BF2G2S1	Fucosylated, Sialylated, Bisected	7.09
2487.849	2487.852	1.13	Hex6dHex1HexNAc5NeuAc1 + Na2	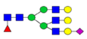	A3F1G3S1	Fucosylated, Sialylated, Triantennary	6.70
2522.888	2522.927	15.10	Hex6HexNAc6NeuAc1 + Na1	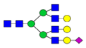	A4G3S1	Sialylated, Tetraantennary	9.28
2537.888	2537.843	17.69	Hex5dHex2HexNAc4NeuAc2 + Na1	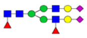	A2F2G2S2	Fucosylated, Sialylated, Biantennary	9.16
2539.904	2539.912	3.39	Hex7dHex1HexNAc6 + Na1	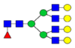	A4F1G4	Fucosylated, Tetraantennary	12.45
2616.892	2616.898	2.37	Hex5dHex1HexNAc5NeuAc2 + Na2	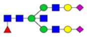	A2BF1G2S2	Fucosylated, Sialylated, Bisected	8.33
2632.886	2632.896	3.80	Hex6HexNAc5NeuAc2 + Na2	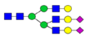	A3G2S2	Sialylated, Triantennary	8.66

2638.873	2638.880	2.43	Hex5dHex1HexNAc5NeuAc2 + Na3	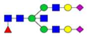	A2BF1G2S2	Fucosylated, Sialylated, Bisected	7.69
2668.946	2668.988	15.77	Hex6dHex1HexNAc6NeuAc1 + Na1	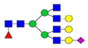	A4F1G3S1	Fucosylated, Sialylated, Tetraantennary	10.84
2778.944	2778.954	3.38	Hex6dHex1HexNAc5NeuAc2 + Na2	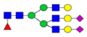	A3F1G3S2	Fucosylated, Sialylated, Triantennary	9.85
2800.926	2800.938	4.32	Hex6dHex1HexNAc5NeuAc2 + Na3	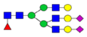	A3F1G3S2	Fucosylated, Sialylated, Triantennary	8.79
3114.004	3114.020	5.20	Hex6dHex1HexNAc5NeuAc3 + Na4	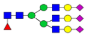	A3F1G3S3	Fucosylated, Sialylated, Triantennary	10.48

Supplementary Table 2. Serum N-glycans with significant differences between benign and DCIS patients. N-glycan compositions are represented by blue squares for N-acetylglucosamine, green circles for mannose, yellow circles for galactose, purple diamonds for sialic acid, and red triangles for fucose.

m/z	Composition	Putative Structure	Oxford Nomenclature	Intensity in DCIS	Fold Change
2179.738	Hex5HexNAc5NeuAc1 + Na2	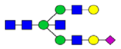	A2BG2S1	Lower	0.826
2413.812	Hex5dHex1HexNAc4NeuAc2 + Na2	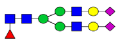	A2G2F1S2	Higher	1.201

Supplementary Table 3. Serum N-glycans with significant associations with age. N-glycan compositions are represented by blue squares for N-acetylglucosamine, green circles for mannose, yellow circles for galactose, purple diamonds for sialic acid, and red triangles for fucose.

m/z	Composition	Putative Structure	Oxford Nomenclature	Intensity Change as Age Increases
1136.396	Hex3HexNAc3 + Na1	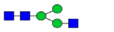	A1	Increases
1339.476	Hex3HexNAc4 + Na1	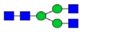	A2	Increases
1444.507	Hex4dHex1HexNAc3 + Na1	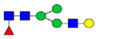	A1G1F1	Decreases
1485.534	Hex3dHex1HexNAc4 + Na1	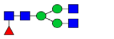	A2F1	Increases
1501.529	Hex4HexNAc4 + Na1	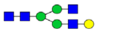	A2G1	Increases
1647.587	Hex4dHex1HexNAc4 + Na1	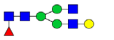	A2G1F1	Decreases
1688.613	Hex3dHex1HexNAc5 + Na1	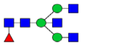	A2BF1	Increases
1757.585	Hex4dHex1HexNAc3NeuAc1 + Na2	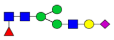	A1F1G1S1	Decreases
1809.639	Hex5dHex1HexNAc4 + Na1	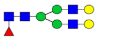	A2F1G2	Decreases
1850.666	Hex4dHex1HexNAc5 + Na1	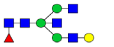	A2BF1G1	Increases
1866.661	Hex5HexNAc5 + Na1	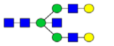	A2BG2	Increases
1955.697	Hex5dHex2HexNAc4 + Na1	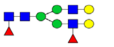	A2F2G2	Increases
2012.719	Hex5dHex1HexNAc5 + Na1	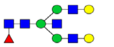	A2BF1G2	Decreases
2100.735	Hex5dHex1HexNAc4NeuAc1 + Na1	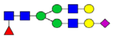	A2F1G2S1	Decreases
2122.717	Hex5dHex1HexNAc4NeuAc1 + Na2	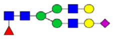	A2F1G2S1	Decreases
2163.743	Hex4dHex1HexNAc5NeuAc1 + Na2	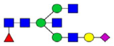	A2BF1G1S1	Increases
2267.754	Hex5HexNAc4NeuAc2 + Na2	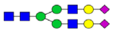	A2G2S2	Increases
2319.809	Hex6HexNAc5NeuAc1 + Na1	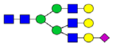	A3G3S1	Increases
2341.791	Hex6HexNAc5NeuAc1 + Na2	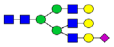	A3G3S1	Increases
2377.851	Hex6dHex1HexNAc6 + Na1	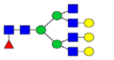	A4F1G3	Increases
2471.854	Hex5dHex2HexNAc5NeuAc1 + Na2	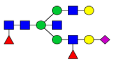	A2BF2G2S1	Decreases
2522.888	Hex6HexNAc6NeuAc1 + Na1	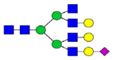	A4G3S1	Increases
2539.904	Hex7dHex1HexNAc6 + Na1	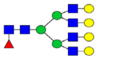	A4F1G4	Increases

2632.886	Hex6HexNAc5NeuAc2 + Na2	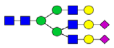	A3G2S2	Increases
Fucosylation	-	-	-	Decreases
Triantennary	-	-	-	Increases
Tetraantennary	-	-	-	Increases

Supplementary Table 4. Serum N-glycans with significant associations with BMI. N-glycan compositions are represented by blue squares for N-acetylglucosamine, green circles for mannose, yellow circles for galactose, purple diamonds for sialic acid, and red triangles for fucose.

m/z	Composition	Putative Structure	Oxford Nomenclature	Intensity Change as BMI Increases
1501.529	Hex4HexNAc4 + Na1	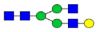	A2G1	Decreases
1809.639	Hex5dHex1HexNAc4 + Na1	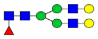	A2F1G2	Decreases
1935.632	Hex6HexNAc3NeuAc1 + Na2	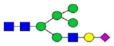	M5A1G1S1	Increases
2163.743	Hex4dHex1HexNAc5NeuAc1 + Na2	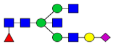	A2BF1G1S1	Increases
2393.846	Hex7HexNAc6 + Na1	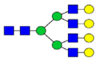	A4G4	Increases
2413.812	Hex5dHex1HexNAc4NeuAc2 + Na2	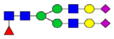	A2F1G2S2	Increases
2435.794	Hex5dHex1HexNAc4NeuAc2 + Na3	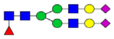	A2F1G2S2	Increases
2487.849	Hex6dHex1HexNAc5NeuAc1 + Na2	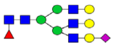	A3F1G3S1	Increases
2668.946	Hex6dHex1HexNAc6NeuAc1 + Na1	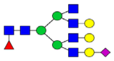	A4F1G3S1	Increases
2778.944	Hex6dHex1HexNAc5NeuAc2 + Na2	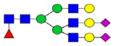	A3F1G3S2	Increases
2800.926	Hex6dHex1HexNAc5NeuAc2 + Na3	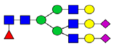	A3F1G3S2	Increases
3114.004	Hex6dHex1HexNAc5NeuAc3 + Na4	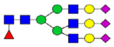	A3F1G3S3	Increases
Biantennary	-	-	-	Decreases
Triantennary	-	-	-	Increases
Tetraantennary	-	-	-	Increases

Supplementary Table 5. Serum N-glycans with significant differences between benign and cancer patients of age 50 - 74 and BMI 18.5 - 24.9. N-glycan compositions are represented by blue squares for N-acetylglucosamine, green circles for mannose, yellow circles for galactose, purple diamonds for sialic acid, and red triangles for fucose.

m/z	Composition	Putative Structure	Oxford Nomenclature	Intensity in Cancer	Fold Change
1850.666	Hex4dHex1HexNAc5 + Na1	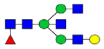	A2BG1F1	Lower	0.726
2163.743	Hex4dHex1HexNAc5NeuAc1 + Na2	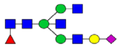	A2BG1F1S1	Lower	0.733
Bisecting	-	-	-	Lower	0.772
